# A rare case of *Bordetella avium* pneumonia complicated by *Raoultella planticola*

**DOI:** 10.1002/ccr3.2800

**Published:** 2020-03-13

**Authors:** Anna Lavrenko, Nataliia Digtiar, Nataliia Gerasymenko, Igor Kaidashev

**Affiliations:** ^1^ Internal Medicine Department No. 3 with Phthisiology Ukrainian Medical Stomatological Academy Poltava Ukraine

**Keywords:** *Bordetella avium*, moxifloxacin, pneumonia, *Raoultella planticola*

## Abstract

*Bordetella avium* pneumonia immunocompromised the patient with subsequent complication by a rare opportunistic *Raoultella planticola* infection, which became a nosocomial pathogen in the healthcare setting.

## Graphical abstract

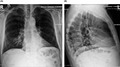

## 1 INTRODUCTION

*Bordetella avium* is a respiratory pathogen isolated from patients with respiratory diseases. The definite pathological mechanism of *B avium* is currently unknown. We report a case of *B avium* pneumonia in a 52‐year‐old man who had regular contact with birds (turkeys, ducks, and geese). The patient was treated by moxifloxacin iv 7 days later, the patient had insufficient clinical effect of the treatment. Repeated sputum culture showed *Raoultella planticola*. We continued the antibiotic therapy for 10 days with clinical success. We suppose that *B avium* pneumonia immunocompromised the patient with subsequent *R planticola* infection. To our knowledge, it was the first case of *B avium* pneumonia complicated by a rare opportunistic pathogen *R planticola*.

*Bordetella avium* (*B avium*) is a Gram‐negative, nonfermentative, strictly aerobic, motile bacterium from the genus *Bordetella*, which has been isolated from patients with respiratory disease such as cystic fibrosis.^1^

In general, *B avium* is a respiratory pathogen causing bordetellosis, a common avian disease in turkeys. Specific adherence and damage to the respiratory epithelia are crucial steps of pathogenesis, but knowledge about the mechanism and the variety of virulence in field strains is limited.^2^

During pathogenesis in turkeys, *B avium* attaches specifically to ciliated respiratory epithelial cells. Ciliated cells are extruded from the epithelium and hyperplasia results in a respiratory epithelium composed of immature cells. Mucus is depleted, and there is eventual infiltration by lymphocytes and macrophages.^3^, ^4^

It was shown that *B avium* does not carry genes that encode pertussis toxin or adenylate cyclase toxin.^5^ *B avium* has yet‐unidentified virulence factors, which may contribute to their ability to cross over from an animal host to an opportunistic human pathogen. In turkey tracheal explants, *B avium* supernatant factors induced apoptosis in ciliated cells.^6^

Recently, it was shown that *B avium* can produce cellulose which may be important for biofilm formation.^7^

Virulence‐associated genes *bvgA*, *fimA,* and *fhaB* (encoded for hemagglutinin) were detected in *B avium* isolates.^8^ The *fhaB* is an important adhesion in the classical Bordetellae, and its expression is regulated by bvgA. Flagella genes play an important role as virulence‐associated factors involved in the motility and the attachment to the host cells.^9^

The abuse of antimicrobials at the poultry farms led to the development of antimicrobial‐resistant bacterial strains. Literature data indicated occurrence of multidrug resistance among turkey *B avium*‐identified isolates with resistance to penicillin, ceftiofur, nalidixic acid, lincomycin, erythromycin, and oxytetracycline.^10^ This resistance may be country‐specific.^11^

Patients with signs and symptoms, clinically consistent with respiratory syndromes caused by *Bordetella,* respond to antibacterial treatment. Human infection with *B avium* may present a challenge for antimicrobial drug treatment due to resistance. The presence of unidentified virulence factors in *B avium* may contribute to their ability to develop into an opportunistic human pathogen from an animal host.^12^

*Raoultella planticola* is a Gram‐negative, aerobic, nonmotile, encapsulated rod‐shaped bacterium belonging to the family Enterobacteriaceae.^13^ Typically considered as a pathogen only in the immunocompromised patients, *R planticola* has been shown to cause various infections in organs such as the lungs, pancreas, skin, liver, prostate, conjunctiva, and gallbladder.^14^, ^15^, ^16^, ^17^

To our knowledge, we present the first case of *B avium* pneumonia in a patient with direct history of attributable environment exposure risk complicated by a rare opportunistic pathogen *Raoultella planticola* (*R planticola*).

## 2 CASE HISTORY AND EXAMINATION

A 52‐year‐old man with a 25‐pack year smoking history and medical history of arterial hypertension, dyscirculatory encephalopathy and chronic pyelonephritis presented to our department with complaints of weakness, fatigue, cough with mucopurulent sputum, and right‐sided moderate chest pain for 5 days. He received paracetamol 500 mg b.i.d., enalapril 10 mg, bisoprolol 10 mg, and acetylsalicylic acid 75 mg. On admission, physical examination revealed a mildly ill‐appearing white male, alert, and oriented, with acrocyanosis.

His vitals were as follows: temperature 40°C, blood pressure 115/70, pulse 73, respiratory rate of 23 breaths per minute, weight 86.0 kg, and height 180.0 cm. The oxygen saturation was 92% on room air.

Auscultation of his lungs revealed rales bilaterally with crackle on the right lower lung field.

Blood count showed white blood cells (WBC) of 5600/mm^3^ with 85% neutrophils and 7% lymphocytes, hemoglobin of 12.1 g/dL, and erythrocyte sedimentation rate 55 mm per hour.

Serum chemistry showed common bilirubin 11.6 μmol/L, creatinine 87 μmol/L, aspartate aminotransferase 32 IU/L, alanine aminotransferase 34 IU/L, total protein 67 g/L, negative HBV and HCV markers, procalcitonin 0.12 μg/L (normal range <.06), and creatinine clearance 106.8 mL/min.

Sputum Gram stain: epithelial cells <10, polymorphonuclear leukocytes >25, gram+ cocci chains rare +1, gram+ cocci pairs few 2+, gram– rods rare 1+, and fungal element rare 1+.

**Figure 1:**
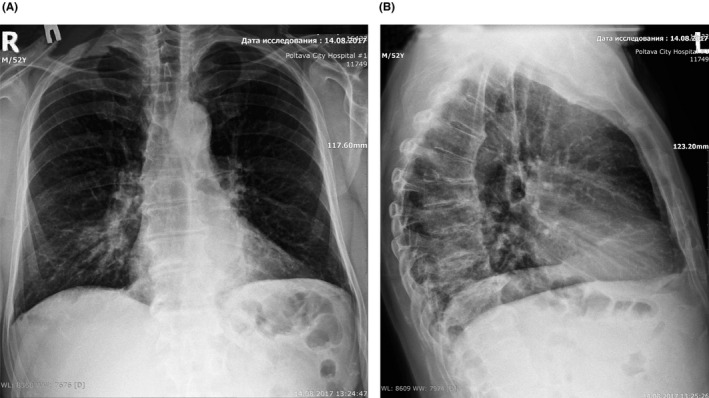
Plane chest radiography of the patient with pneumonia. A, direct projection, B, right side projection

A plain chest radiography on admission showed a right‐sided pleuropneumonia predominantly in the middle lobe (Figure 1).

## 3 DIFFERENTIAL DIAGNOSIS, INVESTIGATIONS, AND TREATMENT

Initially, we suspected bacterial pneumonia. Empirically, ceftriaxone 1000 mg (one time), moxifloxacin 400 mg, and ambroxol were given intravenously. Sputum cultures were prepared and analyzed according to EUCAST recommendation. Sputum culture showed *Metylobacterium mesophilicum*, Viridans group streptococci, and *Candida *spp. *M mesophilicum* isolate was identified as *Bordetella avium* with a sensitivity to most antibiotics (Table 1). Blood culture was sterile. We diagnosed pneumonia caused by *B avium*. Additional investigation revealed that the patient had a private bird farm and regular contact with turkeys, ducks, and geese.

**Table 1:** Antibiotic susceptibility of isolates from pneumonia patients sputum

		Amoxicillin	Amoxicillin/Clavulanic acid	Chloramphenicol	Doxycycline	Co‐trimoxazole	Amikacinum	Ciprofloxacin	Levofloxacin	Moxifloxacin	Cefotaxime	Cefotaxime/Clavulanic acid	Ceftriaxone	Ceftriaxone/Sulbactam	Ceftazidime	Ceftazidime/Clavulanic acid	Cefixime	Cefepime	Lincomycinum
Day 1	*Bordetella avium*	S	S	S	S	S	S	S	S	S	S	S	S	S	S	**–**	**–**	S	–
	*Hemophilus parainfluenzae*	R	R	S	R	R	I	R	I	R	I	S	S	S	S	S	I	I	–
Day 7	*Raoultella planticola*	R	R	S	I	R	S	I	S	S	S	S	S	S	S	S	S	S	–

Note

Abbreviations: –, not done; I, intermediate; R, resistant; S, susceptible.

Seven days after hospitalization, dyspnea, cough, and sputum production were not alleviated. Laboratory analysis was as follows: the WBC count of 4300 mm^3^ (neutrophils 63%, lymphocytes 30%) and erythrocyte sedimentation rate 44 mm/h. A plain chest radiography showed negative dynamics, right‐sided infiltration in S6. An axial chest computed tomography (CT) showed evidence of both sided lower lobe infiltrates with pointed calcificates. Repeated sputum culture (on day 7 after hospitalization) showed *Klebsiella pneumonia*, *Hemophilus parainfluenzae*, and *Rothia (Stomatococcus) mucilanginous*. Isolate of *K pneumoniae* was identified as *Raoultella planticola* with a sensitivity to antibiotics (Table 1). Blood culture was sterile. In serum, no antibodies to HIV were detected. We continued the therapy with moxifloxacin up to 10 days.

## 4 OUTCOME AND FOLLOW‐UP

The patient received intravenous antibiotic for 10 days, with clinical improvement and resolution of pneumonia. He was discharged on nimesulide 100 mg b.i.d. for 5 days.

The patient had follow‐up visit at our clinic after 2 weeks. The blood, urine, and coagulation tests did not reveal any abnormalities.

At the follow‐up visit (12 months), there were no abnormalities in vitals and at a plain chest radiography.

## 5 DISCUSSION

First, an association of human respiratory disease with *B avium* was described by Spilker T. al, (2008) in immunocompromised patients with cystic fibrosis. This report showed that most isolates were identified incorrectly in the initial testing by the referring laboratories, suggesting that further consideration should be given to the possible presence of *Bordetella *spp. in the evolution of sputum culture in the immunocompromised patient.^1^ In our case, we also needed additional verification of a pathogen, identified initially as *Metylobacterium mesophilicum*.

Bordetella will only be detected in sputum cultures when specific technics are used, what makes misidentification of these organisms common using the regular microbial identification methods.^1^ The most reliable method based on the use of PCR with nasopharyngeal aspirates is swabs.^18^

In 2009, Harrington AT, et al described 2 isolates, *B avium* and a novel strain resembling *B avium*, isolated from 2 patients with pneumonia, demonstrating that *B avium* and *B avium*‐like organisms are opportunistic human pathogens. The authors emphasized that although neither patient was conventionally immunocompromised (no HIV, hematologic disorders, or immunosuppressive therapy), each was an elderly person who had pulmonary problems along with other medical conditions, and each belonged to a population typically susceptible to opportunistic infections. Signs and symptoms were clinically consistent with respiratory syndromes caused by *Bordetella*.^12^

To the rare occurrence of human infection, risk factors associated with *B avium* infection are largely deduced from the few reported cases. These include an immunocompromised state and exposure to environmental factors, such as contacts with birds (turkeys, ducks, and geese). Identification of Bordetella species may have serious consequences for treatment in some patients.^19^ Our patient worked at a private bird farm. He took care of birds every day. On admission, he had lymphopenia (7%, 392/mm^3^). We suppose that lymphopenia was due to an immunosuppressive activity of *B avium*. There are no sufficient data about immunosuppressive activity of *B avium*. Nevertheless, other *Bordetella* spp*.* (such as *B pertussis* and *B bronchiseptica*) stimulated immunosuppressive response characterized by increased interleukin‐10 and decreased interferon‐gamma production.^20^, ^21^ The strain of *B avium* was susceptible to many antimicrobials, including moxifloxacin. Such wide susceptibility can be explained by the origin of this strain, from a private bird farm without antimicrobial technologies.

Seven days after moxifloxacin therapy, we did not register clinical success. Primary *K pneumonia* was isolated from the patient's sputum. Later, isolate was identified as *R planticola* with intermediate susceptibility to doxycycline and ciprofloxacin, but susceptible to moxifloxacin. These data support the difficulties in identifying *Klebsiella* strains of clinical origin.^22^

*Raoultella planticola* has traditionally been considered a nonclinical, aquatic, botanical, and soil organism.^13^

Since its initial description, 23 cases of human infections with *R planticola* have been reported.^14^ Thus, *R planticola* may be an emerging pathogen, causing significant infections in multiple different organ systems. *R planticola* should no longer be considered as a harmless environmental organism, but rather as an invasive organism, requiring prompt diagnosis and treatment.

The possible scenarios for *R planticola's* natural course of infection were suggested: (a) trauma infection, (b) nosocomial infection, (c) immunocompromised infection, and (d) enteric fever and bacteremia in immunocompetent patients.^23^

We suggest that our patient had nosocomial *R planticola* infection due to immunocompromising by previous *B avium* pneumonia.

The majority of these patients, infected by *R planticola*, were successfully treated with empirical antibiotics.^24^ However, like *Klebsiella* spp., *R planticola* has the ability to acquire antibiotic resistance.^25^ Recently, there is evidence of carbapenem‐resistant strains of *R planticola*.^26^

Carbapenemase production provided this resistance of *R planticola*.^27^ Our patient had *R planticola* strain resistant to amoxicillin, amoxicillin/clavulanic acid, and co‐trimoxazole. In the literature, there is evidence of successful treatment of such patients with fluoroquinolones.^28^ Thus, in our case, *B avium* and *R planticola* were susceptible to moxifloxacin, and moxifloxacin provided clinical success in pneumonia treatment.

## 6 CONCLUSION

We report, to our knowledge, the first case of *B avium* pneumonia complication by a rare opportunistic pathogen *R planticola* in a patient with direct attributable environment exposure risk—the work at a bird farm. The problems of *B avium* identification still persist. We suppose that *B avium* induced lymphopenia in the patient and immunocompromised him. In this case, *R planticola* became a nosocomial pathogen in the healthcare setting. *R planticola* had susceptibility to many antibiotics with the exception of amoxicillin, clavulanic acid, and co‐trimoxazole in the treatment of pneumonia.

## CONFLICT OF INTEREST

The authors do not have any conflicts of interest to declare.

## AUTHOR CONTRIBUTIONS

AL: involved in data acquisition and manuscript preparation. ND: involved in clinical case management and manuscript preparation. NG: involved in clinical case management and manuscript preparation. IK: involved in concepts, study design, definition of intellectual content, data analysis, manuscript preparation, manuscript editing, and manuscript review.

## ETHICAL APPROVAL

The study has been approved by the appropriate ethics committee and has therefore been performed in accordance with the ethical standards laid down in the 1964 Declaration of Helsinki and its later amendments.
